# Alarming rise in HIV cases in Pakistan: Challenges and future recommendations at hand

**DOI:** 10.1002/hsr2.1450

**Published:** 2023-07-28

**Authors:** Muhammad Aizaz, Farrakh Ali Abbas, Arshad Abbas, Shehroze Tabassum, Emmanuel Ifeanyi Obeagu

**Affiliations:** ^1^ Shandong Provincial Key Laboratory of Animal Resistance Biology College of Life Sciences, Shandong Normal University Jinan Shandong China; ^2^ University of Sargodha Sargodha Pakistan; ^3^ King Edward Medical University Lahore Pakistan; ^4^ Department of Medical Laboratory Science Kampala International University Kampala Uganda

**Keywords:** AIDS, CD4+ T lymphocytes, global challenge, HIV, Pakistan

## Abstract

**Background:**

Human immunodeficiency virus (HIV) is a retrovirus that suppresses the immune system by reducing the CD4+ T lymphocytes level. It has become a global challenge with fast prevalence ratio. Like other developing countries, Pakistan is also struggling for overcoming this viral disease since very first reported case in 1987.

**Aim:**

To update the society on the alarming rise in HIV cases in Pakistan: challenges and future recommendations at hand.

**Materials and Methods:**

The review paper utilized different search engines such pubmed central, scopus, web of science, google scholar etc. to conduct this review paper.

**Results:**

Lack of awareness, low literacy rate, practice of unhygienic equipment in healthcare departments, unstable economy, and unsafe sexual practices are the major factors behind the increasing rate of AIDS in Pakistan.

**Conclusion:**

By regulating healthcare practices and policies, promoting psychological counseling to HIV positive patients, educating the society and minimizing commercial sex practices, Pakistan can overcome this viral disease.

## 1 INTRODUCTION

From the last 40 years, human immunodeficiency virus (HIV) has become a global issue. The researchers studied the pathology of HIV including its etiology, pathogenesis, morphological changes and biochemistry to overcome the immunosuppression caused by it.^1^ HIV (member of *Lentivirus* genus),^2^ has two types HIV‐1 and HIV‐2. HIV‐1 and HIV‐2 have the same basic structures, like presence of three structural genes *gag*, *pol* and *env*, but they both vary in organization of their genome.^3^ Both types of HIV infect the immune system cells and alter the immune response. This effect spreads to the regional lymph node and blood stream.^4^

Primary targets for HIV are CD4+ immune cells. The HIV‐1 variant is the most dominant and when it progresses to its advanced stage, it leads to the development of Acquired Immunodeficiency Syndrome.^5^ Injectable drugs, unsafe blood transfusion, commercial sex and men sex with men and transgenders are the basic sources for the virus spreading.^6^ We can define AIDS as an advanced level of HIV in which CD4+ cells are lesser than 200 per cubic millimeter. Aids results in various infections, cancers and tumors as it is an immunosuppression phenomenon.^7^ According to the *Factsheet* published by *UNAIDS*, the number of affected people has increased up to 38.4 million in 2021 out of which 1.5 million were newly affected in 2021.^8^ HIV is one of the leading causes for mortality worldwide.^9^

**Figure 1:**
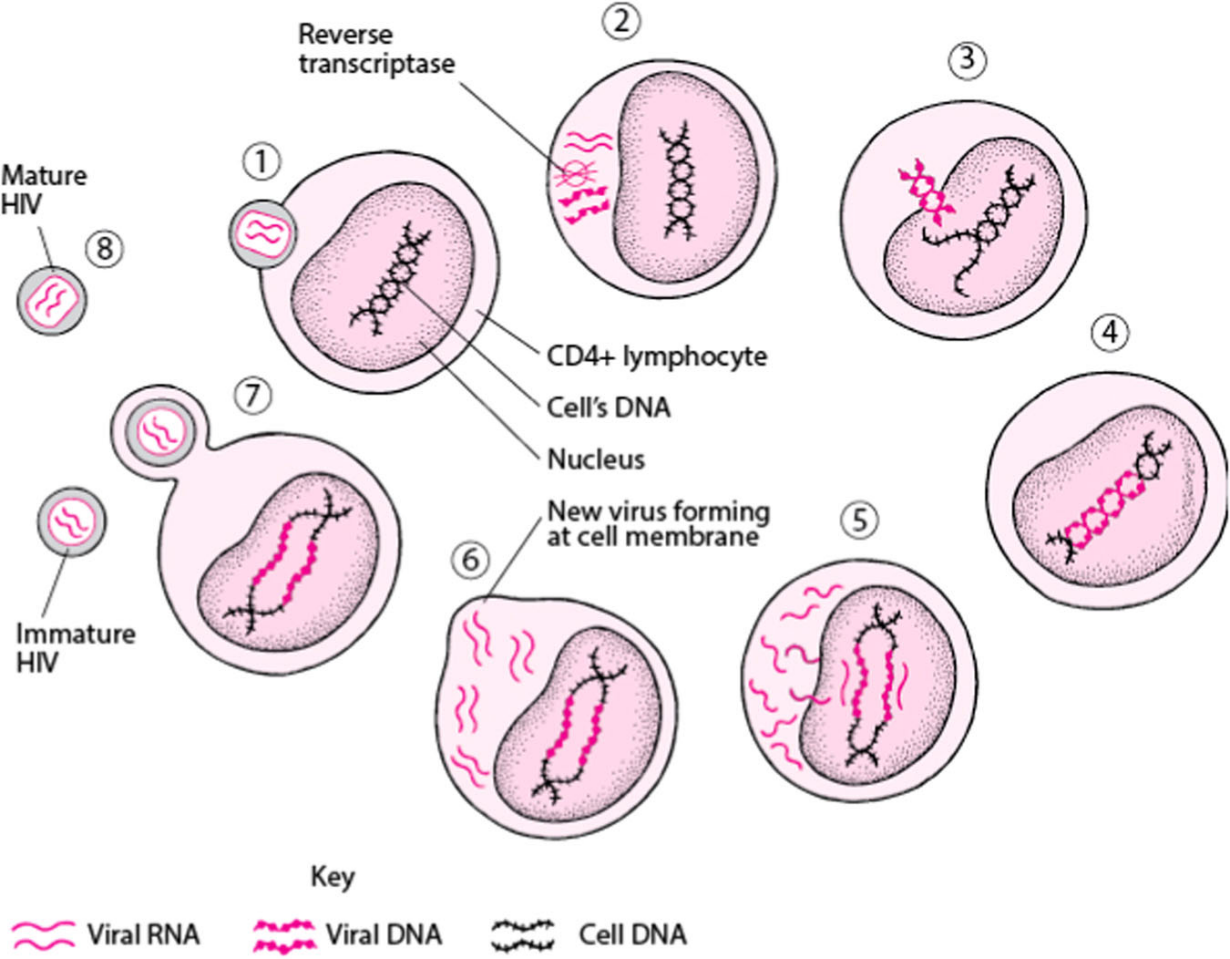
Life cycle of HIV virus.^10^ HIV, human immunodeficiency virus.

Like other viruses, HIV attaches to its target cell i.e. CH4+ lymphocytes and injects its RNA into host cell after penetration. RNA is converted into DNA in a process called as reverse transcription. Viral DNA integrates with host DNA by *integrase* enzyme. After integration, the infected cell DNA produces viral RNA and proteins essential for assembling new HIV. The virus buds it through cell membrane by covering itself with cell membrane fragments. Then it is ejected from the infected CD4+ cells. These immature viruses are matured when HIV protease breaks down the structural proteins in the virus. The life cycle of virus is shown in Figure 1.^10^

A few first aid procedures should be followed while dealing with someone who is suspected of having AIDS. The most crucial action is to get help right away from a trained healthcare expert. Maintaining composure and reassuring the person while waiting for assistance is crucial. You should also control any bleeding, protect yourself by donning gloves and a face mask, avoid sharing personal items that might be contaminated with blood or other bodily fluids, and show support to anyone who may be feeling alone and isolated. When dealing with someone who may have AIDS, getting medical help as soon as you can is essential.^11^ Drugs for treatment of HIV are also formulated on this life cycle. Drugs inhibit the activity of these 3 enzymes i.e. *reverse transcriptase*, *integrase* and *protease*.^10^

## 2 CURRENT STATUS IN PAKISTAN

In 1987, Pakistan came across its very first HIV case which was caused because of unsafe blood transfusion.^12^ In comparison to its neighbors, Pakistan has experienced comparatively few locally infected HIV cases over the past 20 years. In 2004, Injection Drug Users were infected with HIV in remote areas of Larkana (the district of Sindh province) which was very first outbreak of HIV in Pakistan.^13^ In Asia‐Pacific region, Pakistan has the second fastest rate of AIDS breakdown.^14^

Epidemic was discovered in Kot Imrana, a village of tehsil Kot Momin Sargodha in 2018 with the prevalence rate of 1.3%. This was transmitted due to reuse of contaminated needles by quacks. In 2019, this rate was increased up to 13%.^15^ Later in April 2019, 604 children and 135 adults were identified HIV positive.^16^ These infected children have HIV negative parents which shows the horizontal transmission of virus through the use and reuse of contaminated syringe needles in local healthcare facilities.^17^

**Figure 2:**
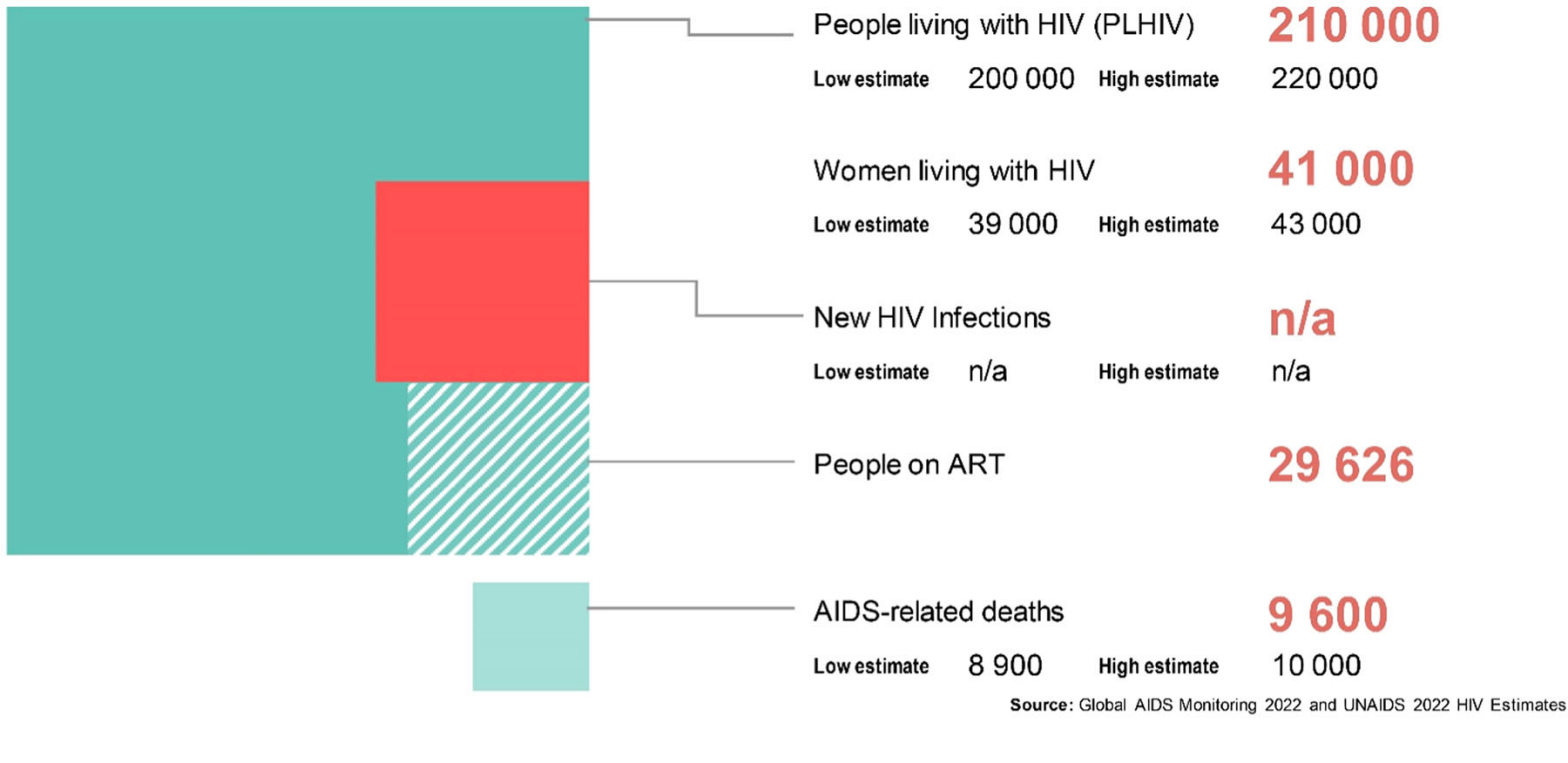
Current Status of HIV in Pakistan.^18^ HIV, human immunodeficiency virus.

According to the Figure 2, the factsheet 2021 published by HIV/AIDS Data Hub for Asia Pacific, Pakistan is having approximately 210,000 people with HIV. A total of 210,000 are adults with 15+ age in which 41,000 are women and 170,000 are men. While the children below 15 years are 4600.^8^

According to the National Aids Control Program (a project of Government of Pakistan), the registered HIV cases are 53,718 out of which 32,972 are receiving antiretroviral therapy (ART) in 51 ART centers which is 61% of registered HIV cases.^18^

## 3 CHALLENGES OF HUMAN IMMUNODEFICIENCY VIRUS

The low literacy rate of Pakistan results in the increasing rate of transmission and haphazardly spreading of HIV because people don't understand the precautionary steps and measures taken by health reforming institutions due to their illiteracy.^19^ Homosexual intercourse, extramarital relationships, unsafe blood transfusion and usage of contaminated syringes result in spreading of HIV.^20^ Concealing HIV of an infected person due to the fear of community prejudicing also results in spreading of HIV.^21^ The unsafe and unlawful sex in prostitution also results in transmission of HIV.^22^ A total of 5.5% HIV prevalence was estimated in the transgender community of Pakistan in 2020.^23^ Healthcare workers are also exposed to the infection due to the lack of guidelines and proper safety equipment.^24^

Poor literacy rates, widespread poverty, and hazardous blood transfusions made Pakistan more assailable to HIV.^25^ The medical professionals and students have major gap in their knowledge and practices.^26^ Due to societal embarrassment and the burden of admitting the reality of extramarital sexual behavior, which embarrasses them in front of their families and even doctors, patients occasionally are reluctant to disclose symptoms at clinics. The growing number of new HIV patients, coupled with the lengthy travel durations to receive medical attention, substantially restrict the accessibility and adherence of ART.^27^

Because HIV is a virus that mutates quickly, developing a vaccine to protect against all HIV mutations is a huge issue.^28^ Currently, the sole therapeutic option is ART. Around 147,851 Pakistanis do not have access to it, and in the last 6 months, 7182 patients missed follow‐up appointments.^21^ Because they cannot be produced in Pakistan and need to be imported, ARTs are short in supply. Another significant issue in global health is HIV medication resistance.^29^

## 4 BURDEN OF HUMAN IMMUNODEFICIENCY VIRUS ON PAKISTAN

HIV cases are increasing every day, as is the mortality toll. According to the National Health Survey as of August 2018, Pakistan had 0.15 million HIV patients, out of which 48% were from Punjab, 38.7% from Sindh, 10% from Khyber Pakhtunkhwa, and 3.3% from Baluchistan.^30^ The fifth Integrated Biological and Behavioral Surveillance Round (2016) had shown a gradually increase in HIV prevalence which was largely caused due to the usage of unsafe and unhygienic needles during drug injecting. Transgenders having sex, male having sex with males and female sex workers also contributed to this prevalence. Latest research indicates that in 2019, 23% of the new infections occurred in injected drugs users, 18% in Men with homosexual relations, 3% in transgenders and 1% in female sex workers.^31^

Young people, or those between the ages of 18 and 30, had a high frequency (19.04%). Males were reported to be more vulnerable to HIV infection.^32^ National AIDS Control Program shows that infection rates among transgender and male sex workers might reach to 60% and 36%, respectively.^30^ It may be note that homosexuals are marking up 5.2% of Pakistan's overall population which is a major contributor to the spread of AIDS. As a result, there are now approximately 85,000 additional AIDS patients in the homosexual community.^21^ Less than 1% of the general population in Pakistan has been estimated to have HIV, which suggests the epidemic is in its concentrated phase. However, the main high‐risk groups are injectable drug users, repatriated migrant laborers, and sex workers.^33^

Diseases like HIV have marginally higher impact in a country facing economic issues, low literacy rate and where people believe in myths and misconceptions instead of proper health protocols.^34^

## 5 CHALLENGES OF HUMAN IMMUNODEFICIENCY VIRUS INFECTIONS FACED BY PATIENTS AND SOCIETY IN PAKISTAN

Regardless of whether it appears ridiculous, the affected must first accept the situation before they can successfully overcome it.^6^ Yet though, Pakistani society has not realized that HIV and AIDS have any link to them. Although there have been changes in trends, there are still many prohibitions and stereotypes around HIV. Especially in rural areas, HIV is viewed as terribly unpleasant. Even talking about this issue is prohibited.^35^ There is very little general knowledge of HIV/AIDS. A study of instructors at schools in the nation's capital, Islamabad, revealed the seriousness of the crisis. A remarkable 60% of the instructors who replied said they believed that HIV was unrelated to our cultural context.^6^

People with medical disorders also suffer from mental health issues like anxiety, depression etc.^36^ Today, more than 350 million individuals worldwide suffer from depression. Depression has a lifetime prevalence of around 3%–17% and is the fourth most common disability globally.^37^

According to recent estimates, roughly 121 million HIV/AIDS‐infected people worldwide suffer from depression.^38^ A total of 32.2% prevalence rate depressions have been noted in HIV positive patients living in Lahore, Pakistan. Surrounding environment, available facilities, age, job history and working history are the factors for depression among HIV patients.^39^

When compared to other parts of Pakistan, Punjab province has higher HIV prevalence due to a number of causes. One cause could be Punjab's increased population density and urbanization, which could increase the virus's ability to spread. In Punjab, cultural and societal traditions may also make it more challenging to openly discuss and handle HIV‐related concerns, which might increase stigma and prejudice against those who are HIV‐positive.^33^ In some parts of Punjab, particularly in rural or distant locations, access to healthcare and preventative services may also be restricted. A greater risk of HIV transmission may be caused by the lack of access to preventative services such condoms, clean syringes for drug users, HIV testing, and counseling.^40^

Many health problems that people with AIDS frequently deal with can have an impact on their general wellbeing. Due to their compromised immune systems, they are more prone to opportunistic infections like pneumonia, tuberculosis, and some cancers. HIV may also affect the brain and nerve system, which can result in neurological conditions including dementia, neuropathy, and seizures. Cardiovascular illness, as well as mental health conditions including depression, anxiety, and drug addiction, are possible health problems for persons with AIDS. In addition, there may be problems with sex desire, vaginal dryness, and erectile dysfunction. AIDS patients often have nutritional deficits, skin concerns, gastrointestinal problems, and oral health problems. A multidisciplinary strategy combining healthcare experts, mental health specialists, and other support services is often necessary for the proper treatment of various health problems. Antiretroviral therapy (ART), drugs to treat opportunistic infections, and counseling and assistance to treat mental health difficulties are all possible forms of treatment. People with AIDS may enhance their quality of life and better manage their illness by addressing these health concerns.^41^, ^42^

Homosexuality is seen as a sin and is prohibited in the Islamic religion and tradition. Conflicts between cultures have resulted as a result in Pakistan, where Muslims make up the majority of the population. In Pakistan, homosexuality is stigmatized, and many LGBTQ+ persons with AIDS experience prejudice and social exclusion.^43^, ^44^

## 6 RECOMMENDATIONS AND CONCLUSION

In light of the alarming situation of HIV/AIDS in Pakistan, it is imperative to take significant measures to prevent the disease from spreading further. The establishment and regular updating of laboratories, dialysis facilities, and dentistry offices, following World Health Organization guidelines, are necessary to minimize the spread of the disease. Moreover, the use of unhygienic equipment during medical procedures should be strongly discouraged. Social awareness campaigns, utilizing various digital, print, and electronic media, are crucial to educating the public about HIV. HIV awareness seminars should be conducted in public and academic institutions to increase knowledge and understanding about the disease. The national and provincial AIDS control programs must strengthen their services, particularly in remote areas, and restructure their efforts to enhance coverage and outreach to targeted populations. Including HIV/AIDS as a chapter in the academic curriculum would help educate students about the disease. Finally, a comprehensive long‐term plan, led by the National AIDS Control Program, is essential to minimize the spread of HIV/AIDS in the country.

To effectively combat the HIV epidemic in Pakistan, several long‐term measures need to be implemented. First and foremost, public knowledge and education on HIV prevention and treatment, especially in marginalized communities, must be increased through targeted awareness campaigns and educational initiatives. Strengthening the healthcare system, making testing and medication more accessible, and providing adequate care for HIV‐positive patients are essential steps. Addressing underlying social and economic issues such as poverty, discrimination, and stigma is also necessary to prevent the spread of HIV. The implementation of policies and initiatives that support social and economic empowerment and reduce the marginalization of at‐risk communities is vital. Finally, collaboration and cooperation with other nations to exchange information, resources, and best practices, as well as to push for more financing for HIV prevention and treatment programs worldwide, is critical. The urgent and essential implementation of these mitigation methods and immediate actions is necessary to control HIV/AIDS in Pakistan, the world's fifth‐most populous nation. The government must take these measures seriously to ensure the wellbeing of its citizens and to minimize the spread of HIV in the country.

In conclusion, owing to a lack of information and education, poverty, and a shoddy healthcare system, HIV continues to be a significant public health problem in Pakistan. However, by putting in place focused interventions and resources, it is feasible to reduce the prevalence of HIV and enhance the quality of life for those who are infected. Raising awareness, promoting safe sexual practices, expanding access to healthcare services, eradicating stigma, and offering HIV‐positive individuals appropriate care and support are just a few of the things that are included in this. Long‐term actions should also be implemented to address the underlying social and economic problems, such as poverty and prejudice, that contribute to the spread of HIV. The support and participation of the Pakistani government, medical experts, and local people will be essential to the success of these measures. Together, we can limit the spread of HIV and enhance the lives of individuals who are infected with the virus by prioritizing initiatives for HIV prevention and treatment.

## AUTHOR CONTRIBUTIONS

**Muhammad Aizaz**: Conceptualization; Investigation; Methodology; Visualization; Writing—original draft. **Farrakh Ali Abbas**: Visualization; Writing—original draft. **Arshad Abbas**: Writing—original draft. **Shehroze Tabassum**: Writing—review & editing. **Emmanuel Ifeanyi Obeagu**: Supervision; Visualization; Writing—review & editing.

## CONFLICT OF INTEREST STATEMENT

The authors declare no conflicts of interest.

## ETHICS STATEMENT

There are no human participants in this article and informed consent is not applicable.

## TRANSPARENCY STATEMENT

The lead author Emmanuel Ifeanyi Obeagu affirms that this manuscript is an honest, accurate, and transparent account of the study being reported; that no important aspects of the study have been omitted; and that any discrepancies from the study as planned (and, if relevant, registered) have been explained.

## Data Availability

No data was used for the research described in the article.
